# 2-Amino-5-methyl­pyridinium 2-carb­oxy­acetate

**DOI:** 10.1107/S1600536810019239

**Published:** 2010-05-29

**Authors:** Madhukar Hemamalini, Hoong-Kun Fun

**Affiliations:** a X-ray Crystallography Unit, School of Physics, Universiti Sains Malaysia, 11800 USM, Penang, Malaysia

## Abstract

In the title mol­ecular salt, C_6_H_9_N_2_^+^·C_3_H_3_O_4_^−^, the cation is essentially planar, with a maximum deviation of 0.010 (3) Å. In the anion, an intra­molecular O—H⋯O hydrogen bond generates an *S*(6) ring and results in a folded conformation. In the crystal, the protonated NH group and the 2-amino group of the cation are hydrogen bonded to the carboxyl­ate O atoms of the anion *via* a pair of N—H⋯O hydrogen bonds, forming an *R* ^2^_2_(8) ring motif. Weak inter­molecular C—H⋯O inter­actions help to further stabilize the crystal structure.

## Related literature

For background to the chemistry of substituted pyridines, see: Pozharski *et al.* (1997 ▶); Katritzky *et al.* (1996 ▶). For related structures, see: Nahringbauer & Kvick (1977 ▶); Feng *et al.* (2005 ▶); Xuan *et al.* (2003 ▶); Jin *et al.* (2005 ▶); Hemamalini & Fun (2010**a* ▶,*b* ▶,c* ▶). For details of hydrogen bonding, see: Jeffrey & Saenger (1991 ▶); Jeffrey (1997 ▶); Scheiner (1997 ▶). For the conformation of the malonate ion, see: Djinović *et al.* (1990 ▶). For hydrogen-bond motifs, see: Bernstein *et al.* (1995 ▶). For bond-length data, see: Allen *et al.* (1987 ▶). For the stability of the temperature controller used in the data collection, see: Cosier & Glazer (1986 ▶).
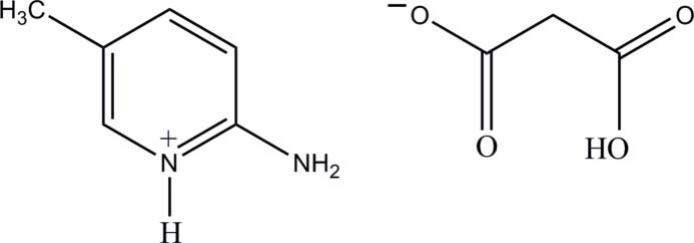


## Experimental

### 

#### Crystal data

C_6_H_9_N_2_^+^·C_3_H_3_O_4_^−^
*M* *_r_* = 212.21
Monoclinic, 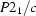

*a* = 3.8082 (13) Å
*b* = 16.963 (5) Å
*c* = 15.372 (5) Å
β = 95.436 (9)°
*V* = 988.6 (5) Å^3^
*Z* = 4
Mo *K*α radiation
μ = 0.11 mm^−1^
*T* = 100 K
0.22 × 0.21 × 0.13 mm

#### Data collection

Bruker APEXII DUO CCD diffractometer
Absorption correction: multi-scan (*SADABS*; Bruker, 2009 ▶) *T*_min_ = 0.975, *T*_max_ = 0.986
8134 measured reflections
2210 independent reflections
1647 reflections with *I* > 2σ(*I*)
*R*_int_ = 0.049

#### Refinement

*R*[*F* ^2^ > 2σ(*F* ^2^)] = 0.058
*wR*(*F* ^2^) = 0.185
*S* = 1.11
2210 reflections
180 parameters
H atoms treated by a mixture of independent and constrained refinement
Δρ_max_ = 0.41 e Å^−3^
Δρ_min_ = −0.35 e Å^−3^

### 

Data collection: *APEX2* (Bruker, 2009 ▶); cell refinement: *SAINT* (Bruker, 2009 ▶); data reduction: *SAINT* (Bruker, 2009 ▶); program(s) used to solve structure: *SHELXTL* (Sheldrick, 2008 ▶); program(s) used to refine structure: *SHELXTL*; molecular graphics: *SHELXTL*; software used to prepare material for publication: *SHELXTL* and *PLATON* (Spek, 2009 ▶).

## Supplementary Material

Crystal structure: contains datablocks global, I. DOI: 10.1107/S1600536810019239/hb5460sup1.cif

Structure factors: contains datablocks I. DOI: 10.1107/S1600536810019239/hb5460Isup2.hkl

Additional supplementary materials:  crystallographic information; 3D view; checkCIF report

## Figures and Tables

**Table 1:** Hydrogen-bond geometry (Å, °)

*D*—H⋯*A*	*D*—H	H⋯*A*	*D*⋯*A*	*D*—H⋯*A*
O3—H1*O*3⋯O1	1.00	1.53	2.475 (3)	157
N1—H1*N*1⋯O2^i^	1.02 (3)	1.65 (3)	2.652 (3)	170 (3)
N2—H1*N*2⋯O4^ii^	0.90 (3)	2.00 (3)	2.886 (3)	171 (3)
N2—H2*N*2⋯O1^i^	0.98 (3)	1.95 (3)	2.924 (3)	177 (3)
C2—H2*A*⋯O3^ii^	0.93 (3)	2.58 (3)	3.470 (3)	159 (2)
C8—H8*A*⋯O2^iii^	0.95 (3)	2.41 (3)	3.304 (3)	158 (3)

Symmetry codes: (i) 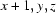; (ii) 
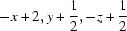
; (iii) 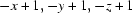.

**Fig. 1.**
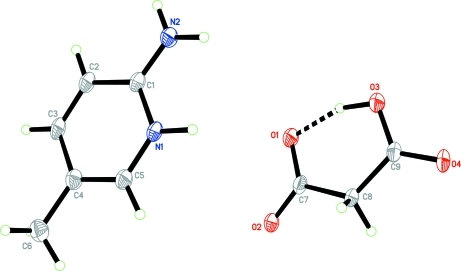
The asymmetric unit of (I). Displacement ellipsoids are drawn at the 50% probability level.

**Fig. 2.**
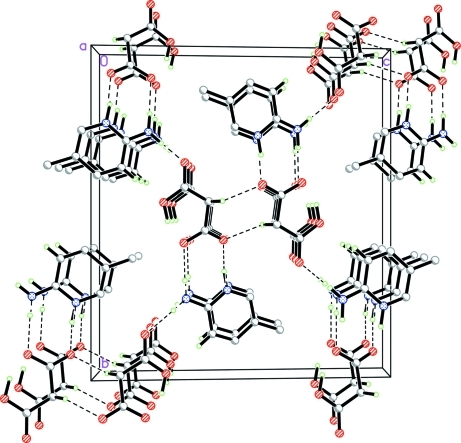
The crystal packing of (I), showing hydrogen-bonded (dashed lines) networks. H atoms not involved in the hydrogen bond interactions are omitted for clarity.

## supplementary crystallographic information

### Comment

Pyridine and its derivatives play an important role in heterocyclic chemistry (Pozharski *et al.*, 1997; Katritzky *et al.*, 1996). They are often involved in hydrogen-bond interactions (Jeffrey & Saenger, 1991; Jeffrey, 1997; Scheiner, 1997). The crystal structures of 2-amino-5-methylpyridine (Nahringbauer & Kvick, 1977), 2-amino-5-methylpyridinium phosphate (Feng *et al.*, 2005), 2-amino-5-methylpyridinium 3-(4- hydroxy-3-methoxyphenyl)-2-propenoate monohydrate (Xuan *et al.*, 2003) and 2-amino-5-methylpyridinium (2-amino-5- methylpyridine)trichlorozincate(II) (Jin *et al.*, 2005) have been reported in the literature. We have recently reported the crystal structures of 2-amino-5-methylpyridinium 3-aminobenzoate (Hemamalini & Fun, 2010*a*), 2-amino-5-methylpyridinium 4-nitrobenzoate (Hemamalini & Fun, 2010*b*) and 2-amino-5-methylpyridinium nicotinate (Hemamalini & Fun, 2010*c*) from our laboratory. In order to study some interesting hydrogen bonding interactions, the synthesis and structure of the title salt is presented here.

The asymmetric unit (Fig. 1) contains one 2-amino-5-methylpyridinium cation and one hydrogen malonate anion. The proton transfer from the one of the carboxyl group oxygen atom (O2) to atom N1 of 2-amino-5-methylpyridine resulted in the widening of C1—N1—C5 angle of the pyridinium ring to 123.3 (2)°, compared to the corresponding angle of 117.4 (3)° in neutral 2-amino-5-methylpyridine (Nahringbauer & Kvick, 1977). The 2-amino-5-methylpyridinium cation is essentially planar, with a maximum deviation of 0.010 (3) Å for atom C4. The bond lengths and angles are normal (Allen *et al.*, 1987).

In the crystal packing (Fig. 2), the protonated N1 atom and the 2-amino group (N2) is hydrogen-bonded to the carboxylate oxygen atoms (O6 and O7) via a pair of intermolecular N1—H1N1···O2 and N2—H2N2···O1 hydrogen bonds forming a ring motif *R*^2^_2_(8) (Bernstein *et al.*, 1995). Atom O3 of the carboxyl group of the hydrogen malonate anions forms an intramolecular O3—H1O3···O1 hydrogen bond with the O atom of the carboxylate group (O1) [with graph-set notation *S(6)*], leading to a folded conformation. A similar intramolecular hydrogen bond has been observed in the crystal structures of benzylammonium hydrogen malonate and 4-picolinium hydrogen malonate (Djinović *et al.*, 1990). The crystal structure is further stabilized by weak C2—H2A···O3 and C8—H8A···O2 (Table 1) hydrogen bonds.

### Experimental

A hot methanol solution (20 ml) of 2-amino-5-methylpyridine (27 mg) and malonic acid (52 mg) were mixed and warmed over a heating magnetic stirrer for a few minutes. The resulting solution was allowed to cool slowly at room temperature and colourless blocks of (I) appeared after a few days.

### Refinement

All H atoms were located from a difference Fourier map and refined freely [C–H = 0.93 (4)–1.04 (4) Å and N–H = 0.89 (3)–0.97 (4) Å]. The hydrogen atom H1O3 was positioned geometrically and refined using a riding model.

### Crystal data

**Table:**

C_6_H_9_N_2_^+^·C_3_H_3_O_4_^−^	*F*(000) = 448
*M**_r_* = 212.21	*D*_x_ = 1.426 Mg m^−^^3^
Monoclinic, *P*2_1_/*c*	Mo *K*α radiation, λ = 0.71073 Å
Hall symbol: -P 2ybc	Cell parameters from 3020 reflections
*a* = 3.8082 (13) Å	θ = 2.4–29.9°
*b* = 16.963 (5) Å	µ = 0.11 mm^−^^1^
*c* = 15.372 (5) Å	*T* = 100 K
β = 95.436 (9)°	Block, colourless
*V* = 988.6 (5) Å^3^	0.22 × 0.21 × 0.13 mm
*Z* = 4	

### Data collection

**Table:**

Bruker APEXII DUO CCD diffractometer	2210 independent reflections
Radiation source: fine-focus sealed tube	1647 reflections with *I* > 2σ(*I*)
graphite	*R*_int_ = 0.049
φ and ω scans	θ_max_ = 27.5°, θ_min_ = 1.8°
Absorption correction: multi-scan (*SADABS*; Bruker, 2009)	*h* = −3→4
*T*_min_ = 0.975, *T*_max_ = 0.986	*k* = −22→21
8134 measured reflections	*l* = −19→19

### Refinement

**Table:**

Refinement on *F*^2^	Primary atom site location: structure-invariant direct methods
Least-squares matrix: full	Secondary atom site location: difference Fourier map
*R*[*F*^2^ > 2σ(*F*^2^)] = 0.058	Hydrogen site location: inferred from neighbouring sites
*wR*(*F*^2^) = 0.185	H atoms treated by a mixture of independent and constrained refinement
*S* = 1.11	*w* = 1/[σ^2^(*F*_o_^2^) + (0.0826*P*)^2^ + 1.1878*P*] where *P* = (*F*_o_^2^ + 2*F*_c_^2^)/3
2210 reflections	(Δ/σ)_max_ < 0.001
180 parameters	Δρ_max_ = 0.41 e Å^−^^3^
0 restraints	Δρ_min_ = −0.35 e Å^−^^3^

### Special details

**Table:**

Experimental. The crystal was placed in the cold stream of an Oxford Cryosystems Cobra open-flow nitrogen cryostat (Cosier & Glazer, 1986) operating at 100.0 (1) K.
Geometry. All s.u.'s (except the s.u. in the dihedral angle between two l.s. planes) are estimated using the full covariance matrix. The cell s.u.'s are taken into account individually in the estimation of s.u.'s in distances, angles and torsion angles; correlations between s.u.'s in cell parameters are only used when they are defined by crystal symmetry. An approximate (isotropic) treatment of cell s.u.'s is used for estimating s.u.'s involving l.s. planes.
Refinement. Refinement of F^2^ against ALL reflections. The weighted R-factor wR and goodness of fit S are based on F^2^, conventional R-factors R are based on F, with F set to zero for negative F^2^. The threshold expression of F^2^ > 2σ(F^2^) is used only for calculating R-factors(gt) etc. and is not relevant to the choice of reflections for refinement. R-factors based on F^2^ are statistically about twice as large as those based on F, and R- factors based on ALL data will be even larger.

### Fractional atomic coordinates and isotropic or equivalent isotropic displacement parameters (Å^2^)

**Table:**

	*x*	*y*	*z*	*U*_iso_*/*U*_eq_	
N1	0.9416 (6)	0.73279 (11)	0.44933 (13)	0.0206 (5)	
N2	1.1470 (7)	0.75544 (13)	0.31461 (14)	0.0244 (5)	
C1	1.0172 (7)	0.78349 (13)	0.38551 (16)	0.0200 (5)	
C2	0.9494 (7)	0.86455 (14)	0.39920 (17)	0.0227 (6)	
C3	0.8223 (7)	0.88786 (13)	0.47473 (17)	0.0225 (5)	
C4	0.7545 (7)	0.83392 (14)	0.54172 (16)	0.0220 (5)	
C5	0.8174 (7)	0.75628 (14)	0.52501 (16)	0.0216 (5)	
C6	0.6322 (9)	0.86073 (17)	0.62714 (19)	0.0287 (6)	
O1	0.3293 (5)	0.58819 (10)	0.30911 (11)	0.0254 (5)	
O2	0.0927 (5)	0.58087 (10)	0.43646 (11)	0.0247 (5)	
O3	0.5960 (6)	0.46992 (10)	0.24783 (12)	0.0288 (5)	
H1O3	0.5045	0.5236	0.2593	0.043*	
O4	0.5733 (6)	0.35450 (10)	0.31494 (12)	0.0305 (5)	
C7	0.2547 (7)	0.55182 (13)	0.37706 (15)	0.0201 (5)	
C8	0.3717 (7)	0.46644 (13)	0.38965 (16)	0.0197 (5)	
C9	0.5161 (7)	0.42551 (14)	0.31344 (16)	0.0221 (5)	
H2A	1.011 (8)	0.8991 (17)	0.3559 (18)	0.019 (7)*	
H3A	0.762 (10)	0.943 (2)	0.485 (2)	0.042 (9)*	
H5A	0.767 (7)	0.7161 (15)	0.5656 (16)	0.011 (6)*	
H6A	0.817 (10)	0.885 (2)	0.661 (2)	0.038 (9)*	
H6B	0.426 (11)	0.899 (2)	0.614 (2)	0.044 (10)*	
H6C	0.532 (10)	0.818 (2)	0.660 (2)	0.048 (10)*	
H8A	0.559 (9)	0.4656 (18)	0.4350 (19)	0.025 (8)*	
H8B	0.173 (9)	0.4314 (19)	0.411 (2)	0.033 (8)*	
H1N1	1.008 (10)	0.676 (2)	0.438 (2)	0.045 (10)*	
H1N2	1.209 (8)	0.7882 (19)	0.273 (2)	0.025 (8)*	
H2N2	1.212 (10)	0.700 (2)	0.311 (2)	0.038 (9)*	

### Atomic displacement parameters (Å^2^)

**Table:**

	*U*^11^	*U*^22^	*U*^33^	*U*^12^	*U*^13^	*U*^23^
N1	0.0273 (12)	0.0096 (9)	0.0251 (10)	0.0002 (8)	0.0036 (8)	0.0015 (7)
N2	0.0371 (14)	0.0120 (10)	0.0251 (11)	0.0017 (8)	0.0072 (9)	0.0023 (8)
C1	0.0234 (14)	0.0118 (11)	0.0246 (12)	−0.0001 (9)	0.0013 (9)	0.0018 (8)
C2	0.0273 (15)	0.0105 (11)	0.0301 (13)	−0.0003 (9)	0.0019 (10)	0.0023 (9)
C3	0.0227 (14)	0.0103 (10)	0.0341 (13)	0.0007 (9)	0.0008 (10)	−0.0011 (9)
C4	0.0222 (14)	0.0165 (11)	0.0273 (12)	0.0008 (9)	0.0022 (10)	−0.0030 (9)
C5	0.0264 (14)	0.0145 (11)	0.0237 (11)	0.0001 (9)	0.0025 (10)	0.0007 (9)
C6	0.0320 (17)	0.0244 (13)	0.0302 (14)	0.0001 (11)	0.0052 (12)	−0.0058 (11)
O1	0.0387 (12)	0.0126 (8)	0.0254 (9)	0.0024 (7)	0.0061 (8)	0.0024 (7)
O2	0.0374 (12)	0.0108 (8)	0.0268 (9)	0.0044 (7)	0.0082 (8)	0.0007 (6)
O3	0.0478 (13)	0.0146 (9)	0.0255 (9)	0.0002 (8)	0.0115 (8)	−0.0007 (7)
O4	0.0496 (14)	0.0108 (8)	0.0326 (10)	0.0014 (8)	0.0112 (9)	−0.0030 (7)
C7	0.0252 (14)	0.0116 (10)	0.0232 (12)	−0.0006 (9)	0.0006 (9)	0.0002 (8)
C8	0.0264 (14)	0.0110 (10)	0.0221 (11)	0.0014 (9)	0.0040 (10)	0.0007 (8)
C9	0.0284 (15)	0.0134 (11)	0.0245 (12)	−0.0015 (9)	0.0028 (10)	−0.0028 (9)

### Geometric parameters (Å, °)

**Table:**

N1—C1	1.356 (3)	C5—H5A	0.96 (3)
N1—C5	1.357 (3)	C6—H6A	0.93 (4)
N1—H1N1	1.02 (4)	C6—H6B	1.03 (4)
N2—C1	1.327 (3)	C6—H6C	0.99 (4)
N2—H1N2	0.89 (3)	O1—C7	1.268 (3)
N2—H2N2	0.97 (4)	O2—C7	1.251 (3)
C1—C2	1.419 (3)	O3—C9	1.317 (3)
C2—C3	1.358 (4)	O3—H1O3	0.9974
C2—H2A	0.93 (3)	O4—C9	1.224 (3)
C3—C4	1.419 (4)	C7—C8	1.522 (3)
C3—H3A	0.97 (4)	C8—C9	1.510 (3)
C4—C5	1.367 (3)	C8—H8A	0.95 (3)
C4—C6	1.505 (4)	C8—H8B	1.04 (4)
			
C1—N1—C5	123.3 (2)	C4—C5—H5A	121.0 (15)
C1—N1—H1N1	114 (2)	C4—C6—H6A	110 (2)
C5—N1—H1N1	122 (2)	C4—C6—H6B	108 (2)
C1—N2—H1N2	120 (2)	H6A—C6—H6B	110 (3)
C1—N2—H2N2	120.6 (19)	C4—C6—H6C	113 (2)
H1N2—N2—H2N2	118 (3)	H6A—C6—H6C	110 (3)
N2—C1—N1	119.2 (2)	H6B—C6—H6C	105 (3)
N2—C1—C2	123.8 (2)	C9—O3—H1O3	106.1
N1—C1—C2	117.0 (2)	O2—C7—O1	125.0 (2)
C3—C2—C1	119.6 (2)	O2—C7—C8	116.2 (2)
C3—C2—H2A	124.1 (18)	O1—C7—C8	118.8 (2)
C1—C2—H2A	116.2 (18)	C9—C8—C7	117.6 (2)
C2—C3—C4	122.4 (2)	C9—C8—H8A	105.0 (18)
C2—C3—H3A	121 (2)	C7—C8—H8A	107.3 (19)
C4—C3—H3A	116 (2)	C9—C8—H8B	107.9 (18)
C5—C4—C3	116.0 (2)	C7—C8—H8B	111.9 (19)
C5—C4—C6	122.0 (2)	H8A—C8—H8B	106 (3)
C3—C4—C6	122.1 (2)	O4—C9—O3	121.6 (2)
N1—C5—C4	121.7 (2)	O4—C9—C8	121.0 (2)
N1—C5—H5A	117.3 (15)	O3—C9—C8	117.3 (2)
			
C5—N1—C1—N2	178.1 (2)	C1—N1—C5—C4	0.9 (4)
C5—N1—C1—C2	−2.1 (4)	C3—C4—C5—N1	0.9 (4)
N2—C1—C2—C3	−178.8 (3)	C6—C4—C5—N1	−177.2 (2)
N1—C1—C2—C3	1.5 (4)	O2—C7—C8—C9	170.6 (2)
C1—C2—C3—C4	0.2 (4)	O1—C7—C8—C9	−10.1 (4)
C2—C3—C4—C5	−1.4 (4)	C7—C8—C9—O4	−170.7 (3)
C2—C3—C4—C6	176.7 (3)	C7—C8—C9—O3	13.0 (4)

### Hydrogen-bond geometry (Å, °)

**Table:**

*D*—H···*A*	*D*—H	H···*A*	*D*···*A*	*D*—H···*A*
O3—H1O3···O1	1.00	1.53	2.475 (3)	157
N1—H1N1···O2^i^	1.02 (3)	1.65 (3)	2.652 (3)	170 (3)
N2—H1N2···O4^ii^	0.90 (3)	2.00 (3)	2.886 (3)	171 (3)
N2—H2N2···O1^i^	0.98 (3)	1.95 (3)	2.924 (3)	177 (3)
C2—H2A···O3^ii^	0.93 (3)	2.58 (3)	3.470 (3)	159 (2)
C8—H8A···O2^iii^	0.95 (3)	2.41 (3)	3.304 (3)	158 (3)

Symmetry codes: (i) *x*+1, *y*, *z*; (ii) −*x*+2, *y*+1/2, −*z*+1/2; (iii) −*x*+1, −*y*+1, −*z*+1.

## References

[bb1] Allen, F. H., Kennard, O., Watson, D. G., Brammer, L., Orpen, A. G. & Taylor, R. (1987). *J. Chem. Soc. Perkin Trans. 2*, pp. S1–19.

[bb2] Bernstein, J., Davis, R. E., Shimoni, L. & Chang, N.-L. (1995). *Angew. Chem. Int. Ed. Engl.* **34**, 1555–1573.

[bb3] Bruker (2009). *APEX2*, *SAINT* and *SADABS* Bruker AXS Inc., Madison, Wisconsin, USA.

[bb4] Cosier, J. & Glazer, A. M. (1986). *J. Appl. Cryst.* **19**, 105–107.

[bb5] Djinović, K., Golič, L. & Leban, I. (1990). *Acta Cryst.* C**46**, 281–286.

[bb6] Feng, H., Sun, C.-R., Li, L., Jin, Z.-M. & Tu, B. (2005). *Acta Cryst.* E**61**, o1983–o1984.

[bb7] Hemamalini, M. & Fun, H.-K. (2010*a*). *Acta Cryst.* E**66**, o623–o624. 10.1107/S1600536810005180 PMC2983539 21580381

[bb8] Hemamalini, M. & Fun, H.-K. (2010*b*). *Acta Cryst.* E**66**, o621. 10.1107/S1600536810005301 PMC2983711 21580379

[bb9] Hemamalini, M. & Fun, H.-K. (2010*c*). *Acta Cryst.* E**66**, o662. 10.1107/S1600536810005970 PMC2983647 21580410

[bb10] Jeffrey, G. A. (1997). *An Introduction to Hydrogen Bonding.* Oxford University Press.

[bb11] Jeffrey, G. A. & Saenger, W. (1991). *Hydrogen Bonding in Biological Structures.* Berlin: Springer.

[bb12] Jin, Z.-M., Tu, B., He, L., Hu, M.-L. & Zou, J.-W. (2005). *Acta Cryst.* C**61**, m197–m199. 10.1107/S0108270105006694 15805621

[bb13] Katritzky, A. R., Rees, C. W. & Scriven, E. F. V. (1996). *Comprehensive Heterocyclic Chemistry II.* Oxford: Pergamon Press.

[bb14] Nahringbauer, I. & Kvick, Å. (1977). *Acta Cryst.* B**33**, 2902–2905.

[bb15] Pozharski, A. F., Soldatenkov, A. T. & Katritzky, A. R. (1997). *Heterocycles in Life and Society.* New York: Wiley.

[bb16] Scheiner, S. (1997). *Hydrogen Bonding. A Theoretical Perspective.* Oxford University Press.

[bb17] Sheldrick, G. M. (2008). *Acta Cryst.* A**64**, 112–122. 10.1107/S0108767307043930 18156677

[bb18] Spek, A. L. (2009). *Acta Cryst.* D**65**, 148–155. 10.1107/S090744490804362X PMC2631630 19171970

[bb19] Xuan, R.-C., Wan, Y.-H., Hu, W.-X., Yang, Z.-Y., Cheng, D.-P. & Xuan, R.-R. (2003). *Acta Cryst.* E**59**, o1704–o1706.

